# Postpancreatectomy diarrhoea: prospective, single-centre longitudinal analysis of incidence, risk factors, management, and impact on quality of life.

**DOI:** 10.1093/bjsopen/zrag017

**Published:** 2026-03-24

**Authors:** Giampaolo Perri, Livia Zornetta, Riccardo Pellegrini, Pietro Rigo, Nicola Canitano, Domenico Bassi, Patrizia Burra, Umberto Cillo, Giovanni Marchegiani

**Affiliations:** Hepato-pancreato-biliary and Liver Transplant Surgery Unit, Department of Surgical, Oncological and Gastroenterological Sciences (DiSCOG), University of Padua, Padua, Italy; Gastroenterology and Multivisceral Transplant Unit Department of Surgical, Oncological and Gastroenterological Sciences (DiSCOG), University of Padua, Padua, Italy; Hepato-pancreato-biliary and Liver Transplant Surgery Unit, Department of Surgical, Oncological and Gastroenterological Sciences (DiSCOG), University of Padua, Padua, Italy; Hepato-pancreato-biliary and Liver Transplant Surgery Unit, Department of Surgical, Oncological and Gastroenterological Sciences (DiSCOG), University of Padua, Padua, Italy; Hepato-pancreato-biliary and Liver Transplant Surgery Unit, Department of Surgical, Oncological and Gastroenterological Sciences (DiSCOG), University of Padua, Padua, Italy; Hepato-pancreato-biliary and Liver Transplant Surgery Unit, Department of Surgical, Oncological and Gastroenterological Sciences (DiSCOG), University of Padua, Padua, Italy; Gastroenterology and Multivisceral Transplant Unit Department of Surgical, Oncological and Gastroenterological Sciences (DiSCOG), University of Padua, Padua, Italy; Hepato-pancreato-biliary and Liver Transplant Surgery Unit, Department of Surgical, Oncological and Gastroenterological Sciences (DiSCOG), University of Padua, Padua, Italy; Hepato-pancreato-biliary and Liver Transplant Surgery Unit, Department of Surgical, Oncological and Gastroenterological Sciences (DiSCOG), University of Padua, Padua, Italy

**Keywords:** pancreatic resection, pancreatoduodenectomy, steatorrhoea, pancreatic insufficiency, pancreatic enzyme, diarrhoea

## Abstract

**Background:**

Postpancreatectomy diarrhoea significantly impairs outcomes and patient’s quality of life. Its origin is multifactorial, extending beyond exocrine pancreatic insufficiency. Even with pancreatic enzyme replacement therapy, persistent postpancreatectomy diarrhoea can lead to malnutrition and inhibit adjuvant treatments. This study aimed to evaluate the incidence, risk factors, management, and impact of postpancreatectomy diarrhoea on quality of life.

**Methods:**

This prospective longitudinal study enrolled patients undergoing pancreatectomy at a single tertiary centre from 2023 to 2025. After surgery (at 7, 30, and 90 days), an adapted systemic therapy-induced diarrhoea assessment tool (STIDAT) questionnaire was used to assess for the presence of postpancreatectomy diarrhoea, patient-reported severity, frequency, medication use, associated symptoms, and quality of life.

**Results:**

A total of 237 patients were included (pancreatoduodenectomy 54%, distal pancreatectomy 35%, total pancreatectomy 11%). Overall, the incidence of postpancreatectomy diarrhoea was 32, 41, and 33% at 7, 30, and 90 days, respectively. Postpancreatectomy diarrhoea was most frequent and severe after total pancreatectomy and pancreatoduodenectomy (62 and 50% at 30 days) and least after distal pancreatectomy (22.0% at 30 days) (*P* < 0.001). The severity of postpancreatectomy diarrhoea correlated with worse quality of life and higher STIDAT scores at all time points. Most patients with postpancreatectomy diarrhoea required pancreatic enzyme replacement therapy, with a median dose of 85 000 lipase units per day by 90 days, and up to 37% also needed antidiarrhoeals. Common 30-day symptoms included urgency (52%), abdominal discomfort (69.4%), and incontinence (18.4%). In multivariable analysis, pancreatic ductal adenocarcinoma, vascular resection, and arterial divestment were independent predictors of moderate-to-severe postpancreatectomy diarrhoea, whereas distal pancreatectomy was protective.

**Conclusion:**

Postpancreatectomy diarrhoea is an impactful complication after pancreatectomy, affecting more than one-third of patients even after correct pancreatic enzyme replacement therapy. Patients undergoing vascular resection and arterial divestment are at higher risk of severe postpancreatectomy diarrhoea, and require tailored postoperative management to reduce its negative effects, which include impaired quality of life.

## Teaser

Postpancreatectomy diarrhoea affects over one-third of patients despite appropriate enzyme replacement, significantly impairing quality of life. This prospective study identified key risk factors for moderate-to-severe diarrhoea, including vascular resection, arterial divestment, and pancreatic ductal adenocarcinoma aetiology. The study findings highlight the need for personalized postoperative management to mitigate the clinical and therapeutic impact of postpancreatectomy diarrhoea.

## Introduction

Pancreatic operations are high-risk procedures burdened by significant and critical postoperative morbidity and mortality^1^. However, advancements in surgical techniques, perioperative management, improved patient selection, and the centralization of treatments at specialized high-volume centres have led to a gradual decrease in major morbidity and mortality rates in recent decades. Despite the improvements, the in-hospital mortality rate after pancreatoduodenectomy (PD) remains between 2 and 5%, and postoperative complications still affect 30–60% of patients^1–3^. The most common complications after pancreatectomy have been defined by the International Study Group for Pancreatic Surgery (ISGPS), and include postoperative pancreatic fistula (POPF), postpancreatectomy pancreatitis and haemorrhage, delayed gastric emptying, and chylous and biliary leaks^4–10^. Additionally, long-term consequences of pancreatectomy have been described widely, including both endocrine and exocrine pancreatic insufficiency (EPI)^11,12^.

A common yet often overlooked complication is postpancreatectomy diarrhoea (PPD), which is typically chronic and multifactorial. Currently, PPD lacks a universal definition but is generally characterized as an increase in stool frequency (≥ 3 bowel movements per day), with altered consistency (Bristol Stool Scale (BSS) types 6–7), occurring within 30 days after surgery^13,14^. The incidence of PPD reported in the literature ranges widely from 20% to over 50%, with variations potentially due to different definitions, follow-up duration, and surgical techniques. If left untreated, diarrhoea can lead to nutritional deficiencies, dehydration, delayed functional recovery, impaired access to adjuvant treatment, and a negative impact on patients’ overall quality of life (QoL)^15–17^. PPD may manifest as watery diarrhoea and/or steatorrhoea, an increased fat excretion in stools. The latter is a cardinal symptom of EPI, resulting from the loss of functional pancreatic tissue during resection^9^. Steatorrhoea, characterized by high-fat, foul-smelling, floating stools, has been studied extensively, especially after total pancreatectomy (TP). A review by Scholten *et al*.^18^ on TP outcomes found that 43.5% of patients reported EPI symptoms; even with pancreatic enzyme replacement therapy (PERT), 23.5% had persistent symptoms and commonly diarrhoea (incidence 0–64%).

Beyond EPI, the complexity of PPD stems from other causes like small intestinal bacterial overgrowth (SIBO) and bile acid malabsorption (BAM), which disrupt regular digestion and absorption^19–21^. Surgical factors, such as extended nerve plexus dissection during PD or distal pancreatectomy (DP) with coeliac axis resection (CAR) can also impair gastrointestinal motility and contribute to the development of PPD^22–24^. Notably, Hirano *et al*.^25^ reported that 62.5% of patients experienced PPD after DP-CAR. The underlying disease, extent of surgical resection, and reconstruction method further influence PPD development^17,19^. Despite the prevalence of PPD, its longitudinal course beyond the immediate postoperative period, associated symptoms, and impact on QoL have not been characterized fully using standardized methods. Identifying early and reliable predictors, particularly for clinically significant diarrhoea, is crucial for developing targeted management strategies and to reduce the clinical consequences of PPD^11,15^.

This aim of this study was to prospectively evaluate the incidence, severity, treatment efficacy, and evolution of PPD, using standardized assessment tools, and related gastrointestinal symptoms, at 7, 30, and 90 days following pancreatic resection. An additional aim was to identify preoperative and intraoperative factors that could predict the development of moderate-to-severe PPD.

## Methods

### Study design and patient inclusion

This was a single-centre, prospective observational study conducted at Padua University Hospital. Adult patients (aged > 18 years) who underwent PD, DP or TP for any indication between 1 January 2023 and 30 June 2025 were included consecutively. Exclusion criteria were: pre-existing diagnosis of inflammatory bowel disease, presence of a permanent stoma, a history of partial or total gastrectomy or colectomy, withdrawal of informed consent, and inability to provide consent or to complete questionnaires. Approval of data collection and analysis for this study was obtained from local ethical committee in Padua, Italy. Signed informed consent was obtained from all participants. The STROBE checklist is provided in *Table S1*.

### Preoperative care and intraoperative setting

Preoperative care followed established institutional protocols. Data collected included demographics, indication for surgery, presence of diarrhoea, history of weight loss, and current medication use, particularly focusing on PERT or antidiarrhoeal medications.

Pancreatic resections (PD, DP, TP) were performed according to institutional standards by experienced pancreatic surgeons. During PD, pancreatic texture was classified as hard or soft^26^, and the main pancreatic duct calibre was measured in millimetres. Subsequently, the fistula risk score (FRS) was calculated^27^. The reconstruction was usually carried out through a pancreaticojejunal, hepaticojejunal, and gastrojejunal anastomosis using a Child single loop. Transanastomotic externalized stents (ETSs) were placed selectively in high-risk patients based on FRS score, and in general in patients with a main pancreatic duct diameter of ≤ 3 mm^28^. Lymphadenectomy procedures were performed based on ISGPS guidelines^29^. Standard lymphadenectomy during PD included the removal of lymph node stations 5, 6, 8a, 12b1, 12b2, 12c, 13a, 13b, 14a (right lateral side), 14b (right lateral side), 17a, and 17b. For DP, standard lymphadenectomy involved stations 10, 11, and 18. Lymph node station 9 was resected only when tumours were confined to the body of the pancreas. Extended lymphadenectomy during PD, involving nodes along the left side of the superior mesenteric artery (SMA) and around the coeliac trunk, splenic artery or left gastric artery, was undertaken only when indicated clinically. Periarterial divestment, defined as meticulous dissection along the vessel sheath when SMA involvement is suspected, was performed if deemed clinically necessary and was noted in the records^30^. Additional intraoperative details, such as duration of operation, blood loss, vascular resections (either venous or arterial), and specific reconstruction details, were recorded in the electronic medical records.

### Postoperative care

PERT was not prescribed routinely to all patients after surgery, but rather initiated based on clinical presentation. According to institutional protocol, PERT was started in the presence of symptoms suggestive of EPI, particularly steatorrhoea. After PD and DP, enzyme supplementation was therefore symptom-driven, whereas PERT was administered universally after TP, as complete exocrine insufficiency is inevitable in this setting. The PERT dose (lipase units (LU) per meal/snack) was adjusted based on clinical response, aiming to improve stool consistency and reduce the frequency of bowel movements. If watery diarrhoea persisted—defined in the protocol as more than five episodes per day, especially postprandially—loperamide was introduced. The initial dose was 4 mg, with an additional 2 mg permitted after each subsequent loose stool, up to a maximum daily dose of 16 mg.

### Diarrhoea assessment and questionnaires

All patients had outpatient follow-up visits or received telephone calls at 7, 30, and 90 days after surgery. During follow-up, a physician undertook a standardized clinical evaluation, assessing patients’ self-report of experiencing diarrhoea, number of diarrhoeal episodes, stool consistency recorded using the BSS, and presence of associated symptoms, such as urgency, abdominal discomfort, and incontinence. Diarrhoea was defined as a minimum of three bowel movements per day, each of these classified as having an altered consistency (BSS type 6 or 7)^31,32^. Faecal urgency was defined by the sudden need to rush to the bathroom to empty one’s bowel, whereas faecal incontinence was described as the unintentional loss of solid or liquid stool^33,34^. Steatorrhoea was defined clinically as patient-reported pale, greasy, foul-smelling and floating stools, occurring within the 7 days preceding the assessment. No quantitative faecal fat measurement or faecal elastase 1 testing was performed as part of the study protocol^35^. Additionally, the use of PERT and antidiarrhoeal medications in the postoperative setting was evaluated. At each follow-up point (7, 30, and 90 days), participants completed a structured questionnaire (*Appendix S1*) that focused on bowel habits experienced over the preceding 7 days, specifically exploring the presence of PPD. This questionnaire was adapted from the validated systemic therapy-induced diarrhoea assessment tool (STIDAT), originally developed and validated by Lui *et al*.^36^ to assess chemotherapy-induced diarrhoea. The STIDAT evaluates multiple dimensions: frequency, urgency, incontinence, abdominal pain/spasms, and impact on QoL. QoL was assessed according to the influence of diarrhoea on the patient’s social and family life, mood, ability to undertake work or daily activities, and energy levels^36^. Although the STIDAT has not been validated specifically for this purpose and lacks specific items for steatorrhoea, its use in this study was considered for exploratory reasons. Additionally, information from the STIDAT was complemented by direct assessments of patient-reported severity and the BSS. Diarrhoea severity was categorized as mild, moderate or severe based on a direct patient-reported question concerning the subjective impact of bowel symptoms on daily life. The STIDAT questionnaire has been readapted and modified, inserting PERT among the drugs used. The adapted questionnaire comprises 12 questions exploring bowel habit frequency, consistency (via BSS recall), use and perceived efficacy of PERT and antidiarrhoeals, urgency, incontinence, and abdominal discomfort. Additionally, five QoL items were rated on an 11-point numerical scale from 0 (no impact) to 10 (extreme impact). A total QoL score (from 0 to 50) was calculated, with lower scores indicating better QoL and fewer symptoms affecting overall well-being. The STIDAT scoring algorithm yields a score (*Appendix S2*), with values of ≥ 1.1 indicating diarrhoea, with severity ranges defined as: 1.1–2.0 (mild), 2.0–3.0 (moderate), and > 3.0 (severe)^36^.

### Endpoints

The primary endpoints were PPD incidence, characteristics (frequency, consistency, associated symptoms), and severity (patient-reported impact and STIDAT score) at 7, 30, and 90 days following PD, SP, and TP. Secondary endpoints included impact of PPD on QoL, and identification of preoperative and intraoperative predictors of developing moderate-to-severe forms (based on patient-reported impact) at 30 and/or 90 days.

### Statistical analysis

Continuous variables are expressed as mean(standard deviation, s.d.) or median (interquartile range, i.q.r.), and were compared using the independent-samples *t* test or the Mann–Whitney *U* test, as appropriate. Categorical variables, expressed as numbers with percentages, were analysed using the χ^2^ test, or Fisher’s exact test in the event of expected small frequencies. All the tests were two-tailed. *P* < 0.050 was considered statistically significant. Variables missing at random were considered acceptable for exclusion without imputation with a threshold of ≤ 10% in the complete-case analysis. Possible associations between demographic/clinical factors and moderate/severe diarrhoea at 30 and/or 90 days were evaluated using univariable and multivariable logistic regression modelling, with results expressed as odds ratios (ORs) with 95% confidence intervals Moderate/severe diarrhoea at either 30 or 90 days was selected as the dependent variable owing to its clinical relevance (as opposed to mild diarrhoea in general or moderate/severe diarrhoea only on postoperative day 7). Clinical factors with *P* < 0.050 in univariable analysis and those that had potential clinical importance were included in the model. Statistical analyses were undertaken using Stata^®^ version 14 for Windows^®^ (StataCorp, College Station, TX, USA). Figures were created with BioRender.com.

## Results

### Patient cohort

During the study period, a total of 265 patients were assessed for eligibility, and 237 were finally included (*Fig. S1*). *Table 1* summarizes the baseline demographics, preoperative clinical features, and surgical details of the study cohort. The median age was 67 (i.q.r. 59–76) years, and 128 patients (54%) were women. Before surgery, 89 patients (37.6%) reported weight loss, 43 (18.1%) experienced diarrhoea, and 42 (17.7%) were using PERT. Only four (1.7%) used chronic antidiarrhoeal medication before operation. Operations performed were PD (129, 54.4%), DP (82, 34.6%), and TP (26, 11.0%). Among patients who underwent PD, the pylorus was preserved in 104 (80.6%), and ETSs were used in 53 (41.1%). The median FRS was 3 (i.q.r. 2–5). The spleen was preserved in 12.2% of patients undergoing DP and 23.1% of those having TP. Across the entire cohort, venous resection was performed in 25 patients (10.5%), arterial resection in 4 (1.7%), and SMA divestment in 15 (6.4%). A median of 23.5 lymph nodes was harvested, with extended lymphadenectomy undertaken in 29 patients (12.2%).

**Table 1:** Demographics, preoperative features, surgical details, and postoperative outcomes at discharge

	No of patients* (*n* = 237)
Age (years), median (i.q.r.)	67 (59–76)
**Sex**	
Male	109 (46.0%)
Female	128 (54.0%)
Preoperative weight loss	89 (37.6%)
Preoperative diarrhoea	43 (18.1%)
Preoperative use of PERT	42 (17.7%)
Preoperative use of antidiarrhoeals	4 (1.7%)
**Histological diagnosis**	
PDAC	132 (55.7%)
Periampullary neoplasm	34 (14.3%)
Cystic neoplasm	31 (3.1%)
pNET	27 (11.4%)
Other	13 (5.2%)
**Type of surgery**	
PD	129 (54.4%)
DP	82 (34.6%)
TP	26 (11.0%)
Pylorus preservation (if PD)	104 (80.6%)
Spleen preservation (if DP/TP)	16 (14.8%)
Venous resection	25 (10.5%)
Arterial resection	4 (1.7%)
Arterial divestment	15 (6.4%)
Fistula risk score, median (i.q.r.)	3 (2–5)
Externalized transanastomotic stent (if PD)	53 (41.1%)
Extended lymphadenectomy	29 (12.2%)
No. of lymph nodes harvested, median (i.q.r.)	23.5 (16.3–23)
Diarrhoea during hospital stay	75 (31.6%)
Length of hospital stay (days), median (i.q.r.)	9 (6–15)
**Postoperative complications**	
Postoperative pancreatic fistula	45 (19%)
Postpancreatectomy acute pancreatitis	23 (9.7%)
Postpancreatectomy haemorrhage	57 (24%)
Delayed gastric emptying	32 (13.5%)
Chyle leak	16 (6.8%)
**Clavien–Dindo complication grade**	
I	39 (16.5%)
II	146 (61.6%)
IIIa	24 (10.1%)
IIIb	16 (6.8%)
IVa	6 (2.5%)
IVb	6 (2.5%)
V	0 (0%)
Use of PERT at discharge	105 (44.3%)
PERT dose (LU/day), median i.q.r.)	50 000 (0–60 000)
Use of antidiarrhoeals at discharge	10 (4.2%)

^*^Values are *n* (%) unless otherwise stated. i.q.r., Interquartile range; PERT, pancreatic enzyme replacement therapy; PDAC, pancreatic ductal adenocarcinoma; pNET, pancreatic neuroendocrine tumour; PD, pancreatoduodenectomy; DP, distal pancreatectomy; TP, total pancreatectomy; LU, lipase international units.

Early postoperative outcomes are reported in *Table 1*. In total, 75 patients (31.6%) experienced diarrhoea during the hospital stay. With regard to postoperative complications, POPF was significantly less frequent (10.2 *versus* 25.2%; *P* = 0.004) and chyle leak significantly more common (13.3% *versus* 2.2%; *P* < 0.001) in patients experiencing diarrhoea (*Table S2*). The median duration of hospital stay was 10 (i.q.r. 7–16.5) days for patients who developed diarrhoea during the hospital stay, compared with 8 (6–14.8) days for those who did not (*P* = 0.364). At discharge, 105 patients (44.3%) used PERT with a median dose of 50 000 LU/day, and 10 patients (4.2%) were prescribed antidiarrhoeals.

### Postoperative diarrhoea: incidence, characteristics, and severity

**Fig. 1:**
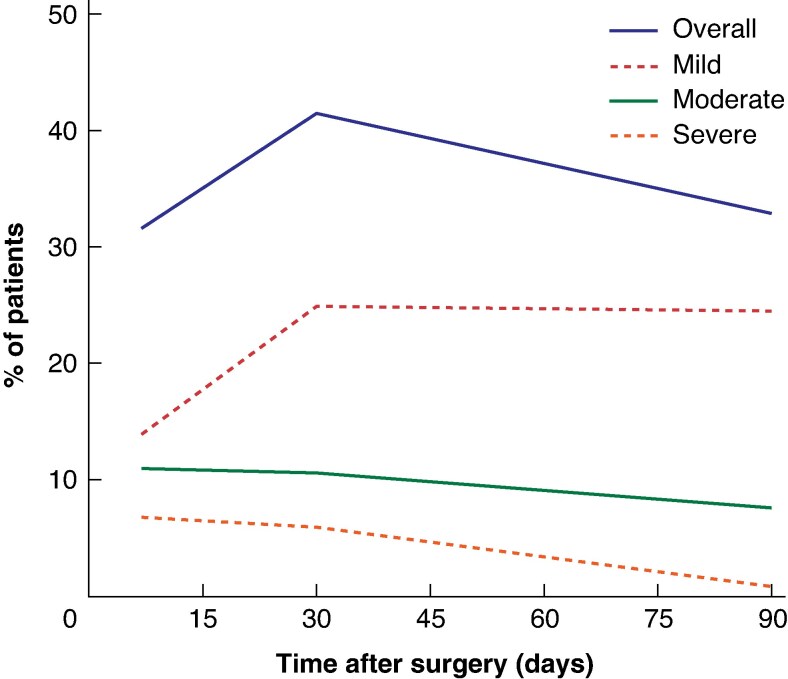
Temporal trends in incidence and severity of diarrhoea

**Fig. 2:**
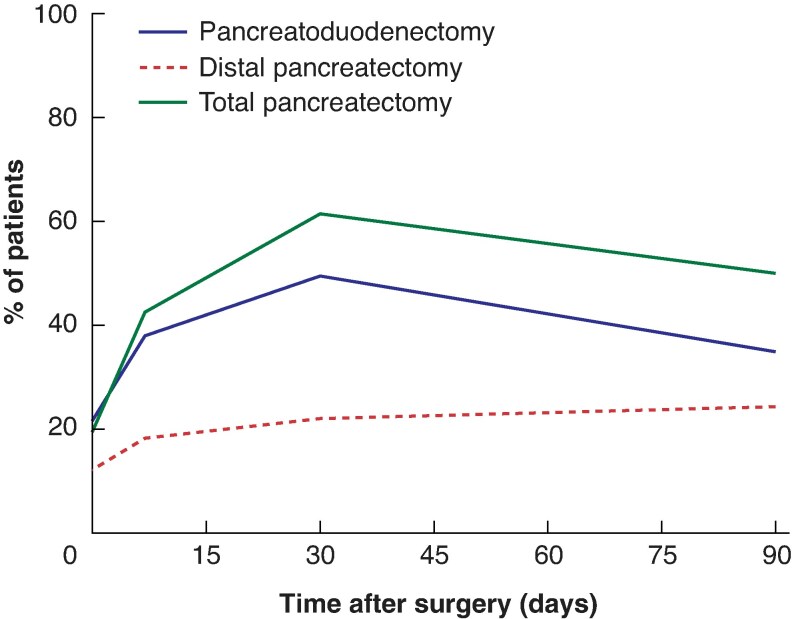
Temporal trends in incidence of diarrhoea by type of surgical procedure

**Fig. 3:**
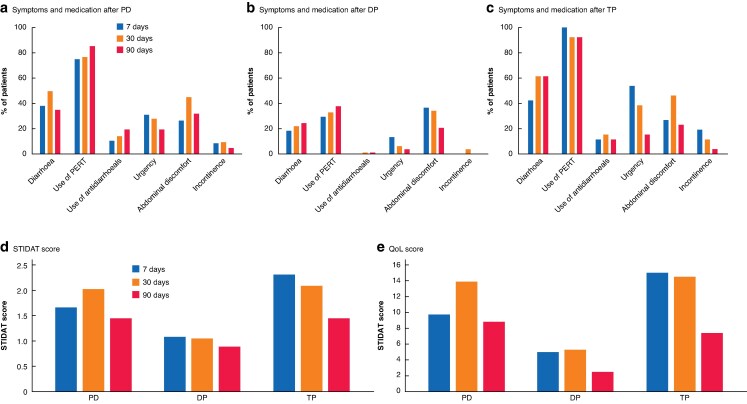
QoL and STIDAT scores, associated symptoms, and medication use categorized by type of surgery Symptoms and medication use after **a** pancreatoduodenectomy (PD), **b** distal pancreatectomy (DP), and **c** total pancreatectomy (TP); **d** systemic therapy-induced diarrhoea assessment tool (STIDAT) and **e** quality-of-life (QoL) scores after each type of surgery. PERT, pancreatic enzyme replacement therapy.

Outcomes for all patients and for those with diarrhoea at 7, 30, and 90 days are shown in *Table 2*. A detailed analysis of postoperative outcomes based on patient-reported diarrhoea severity at 7, 30, and 90 days is provided in *Table 3*. *Table 4* details the incidence of PPD at 7, 30, and 90 days stratified by type of surgical procedure. *Figure 1* illustrates the temporal trends of diarrhoea incidence and severity at 7, 30, and 90 days, and *Fig. 2* displays the temporal trends of diarrhoea incidence according to surgical procedures at the same time points. *Figure 3* shows the incidence of diarrhoea and associated symptoms, medications used, as well as STIDAT and QoL scores, categorized by type of surgical procedure. Finally, *Fig. S2* illustrates the temporal evolution of PPD from the preoperative baseline to 90 days after surgery.

Outcomes 7 days after surgery

**Table 2:** Comparisons of QoL and symptoms in patients with and without diarrhoea at 7, 30 and 90 days

	All patients	Patients without diarrhoea	Patients with diarrhoea	P*
**7 days after surgery**	*n* = 237	*n* = 162	*n* = 75	
STIDAT score, mean(s.d.)	1.6(1.8)	0.5(0)	3.2(1.2)	< 0.001†
QoL score, mean(s.d.)	9.2(14.7)	0(0)	29.2(9.9)	< 0.001†
Faecal incontinence	16 (6.8%)	0 (0%)	16 (21.3%)	0.002
Abdominal discomfort	71 (30%)	50 (30.8%)	21 (28.0%)	0.810
Urgency	65 (27.4%)	0 (0%)	65 (86.7%)	< 0.001
Use of antidiarrhoeals	16 (6.8%)	0 (0%)	16 (21.3%)	0.002
Use of PERT	159 (67.1%)	92 (55.1%)	67 (89.3%)	0.193
PERT dose (LU/day), median (i.q.r.)	50 000 (0–60 000)	35 000 (0–60 000)	50 000 (30 000–65 000)	0.131‡
Stool consistency, median (i.q.r.)	4 (4–6)	4 (3–4)	6 (6–6)	< 0.001‡
No. of diarrhoeal episodes, median (i.q.r.)	1 (0–3)	0 (0–0)	4 (3–6)	< 0.001‡
**30 days after surgery**	*n* = 237	*n* = 139	*n* = 98	
STIDAT score, mean(s.d.)	1.8(1.6)	0.6(0.2)	3.4(1.2)	< 0.001†
QoL score, mean(s.d.)	11.0(13.8)	1.8(3.3)	24.0(12.4)	< 0.001†
Faecal incontinence	18 (7.6%)	0 (0%)	18 (18.4%)	< 0.001
Abdominal discomfort	98 (41.4%)	30 (21.0%)	68 (69.4%)	< 0.001
Urgency	51 (21.5%)	0 (0%)	51 (52.0%)	< 0.001
Use of antidiarrhoeals	23 (9.7%)	0 (0%)	23 (23.4%)	< 0.001
Use of PERT	150 (63.3%)	55 (39.6%)	95 (96.9%)	< 0.001
PERT dose (LU/day), median (i.q.r.)	50 000 (0–80 000)	10 000 (0–50 000)	80 000 (50 000–108 000)	< 0.001‡
Stool consistency, median (i.q.r.)	6 (4–6)	4 (4–5)	6 (6–6)	< 0.001‡
No. of diarrhoeal episodes, median (i.q.r.)	0 (0–3)	0 (0–0)	3 (3–5)	< 0.001‡
**90 days after surgery**	*n* = 237	*n* = 159	*n* = 78	
STIDAT score, mean(s.d.)	1.3(1.2)	0.6(0.2)	2.8(0.9)	< 0.001†
QoL score, mean(s.d.)	6.6(10.6)	1.3(3.3)	17.3(12.2)	< 0.001†
Faecal incontinence	7 (3%)	1 (0.5%)	6 (7.7%)	0.006
Abdominal discomfort	64 (27%)	24 (15.0%)	40 (51.3%)	< 0.001
Urgency	32 (13.5%)	1 (0.5%)	31 (39.7%)	< 0.001
Use of antidiarrhoeals	29 (12.2%)	0 (0%)	29 (37.2%)	< 0.001
Use of PERT	155 (65.4%)	81 (50.9%)	74 (94.9%)	< 0.001
PERT dose (LU/day), median (i.q.r.)	50 000 (0–80 000)	20 000 (0–60 000)	85 000 (60 000–120 000)	< 0.001‡
Stool consistency, median (i.q.r.)	4 (4–6)	4 (4–4)	6 (6–6)	< 0.001‡
No. of diarrhoeal episodes, median (i.q.r.)	0 (0–3)	0 (0–0)	3 (3–3)	< 0.001‡

Values are *n* (%) unless otherwise stated. QoL, quality of life; s.d., standard deviation; i.q.r., interquartile range; STIDAT, systemic therapy-induced diarrhoea assessment tool; PERT, pancreatic enzyme replacement therapy; LU, lipase international units. *χ^2^ test or Fisher’s exact test, except †independent-samples *t* test and ‡Mann–Whitney *U* test.

**Table 3:** QoL and symptoms based on patient-reported diarrhoea severity at 7, 30, and 90 days

	Patient-reported diarrhoea			P*
	Mild	Moderate	Severe	
**7 days after surgery**	*n* = 33	*n* = 26	*n* = 16	
STIDAT score, mean(s.d.)	2.7(0.2)	4.2(0.5)	5.9(0 .9)	< 0.001†
QoL score, mean(s.d.)	21.0(4.6)	31.4(5.9)	42.0(6.7)	< 0.001†
Faecal incontinence	0 (0%)	6 (23.1%)	10 (62.5%)	< 0.001
Abdominal discomfort	7 (21.2%)	7 (26.9%)	7 (43.8%)	0.825
Urgency	23 (69.7%)	26 (100%)	16 (100%)	< 0.001
Use of antidiarrhoeals	0 (0%)	10 (38.5%)	6 (37.5%)	< 0.001
Use of PERT	25 (69.7%)	26 (100%)	16 (100%)	0.473
PERT dose (LU/day), median (i.q.r.)	40 000 (8000–58 000)	70 000 (45 000–103 000)	60 000 (30 000–60 000)	0.178‡
Stool consistency, median (i.q.r.)	6 (6–6)	6 (6–6)	6 (6–7)	< 0.001‡
No. of diarrhoeal episodes, median (i.q.r.)	3 (2.3–3)	5.5 (4–6)	10 (7–10)	< 0.001‡
**30 days after surgery**	*n* = 59	*n* = 25	*n* = 14	
STIDAT score, mean(s.d.)	2.6(0.4)	4.1(0.5)	5.5(0.4)	< 0.001†
QoL score, mean(s.d.)	16.9(8.2)	31.3(10.0)	40.6(6.1)	< 0.001†
Faecal incontinence	2 (3.4%)	7 (28%)	9 (64%)	< 0.001
Abdominal discomfort	42 (71.2%)	15 (60%)	11 (78.6%)	< 0.001
Urgency	17 (28.8%)	21 (84%)	13 (92.9%)	< 0.001
Use of antidiarrhoeals	2 (3.4%)	13 (52%)	8 (57.2%)	< 0.001
Use of PERT	56 (94.9%)	25 (100%)	14 (100%)	< 0.001
PERT dose (LU/day), median (i.q.r.)	60 000 (50 000–80 000)	100 000 (80 000–150 000)	100 000 (80 000–135 000)	< 0.001‡
Stool consistency, median (i.q.r.)	6 (6–6)	6 (6–6)	6.5 (6–7)	< 0.001‡
No. of diarrhoeal episodes, median (i.q.r.)	3 (3–3)	5 (4–5)	6 (6–8)	< 0.001‡
**90 days after surgery**	*n* = 58	*n* = 18	*n* = 2	
STIDAT score, mean (s.d.)	2.4(0.5)	4.0(0.4)	5.0(0.4)	< 0.001†
QoL score, mean(s.d.)	12.6(9.6)	30.4(7.4)	37.5(3.5)	< 0.001†
Faecal incontinence	2 (3.45%)	3 (16.6%)	1 (50%)	< 0.001
Abdominal discomfort	24 (41.4%)	15 (83.3%)	1 (50%)	< 0.001
Urgency	13 (22.4%)	16 (88.9%)	2 (100%)	< 0.001
Use of antidiarrhoeals	14 (23.1%)	13 (72.2%)	2 (100%)	< 0.001
Use of PERT	54 (93.1%)	18 (100%)	2 (100%)	< 0.001
PERT dose (LU/day), median (i.q.r.)	80 000 (50 000–100 000)	105 000 (100 000–150 000)	145 000 (118 000–173 000)	< 0.001‡
Stool consistency, median (i.q.r.)	6 (6–6)	6 (6–6)	7 (7–7)	< 0.001‡
No. of diarrhoeal episodes, median (i.q.r.)	3 (3–3)	4 (3–4)	5 (4.5–5.5)	< 0.001‡

Values are *n* (%) unless otherwise stated. QoL, quality of life; s.d., standard deviation; i.q.r., interquartile range; STIDAT, systemic therapy-induced diarrhoea assessment tool; PERT, pancreatic enzyme replacement therapy; LU, lipase international units. *χ^2^ or Fisher’s exact test, except †independent-samples *t* test and ‡Mann–Whitney *U* test.

**Table 4:** Incidence of postoperative diarrhoea at 7, 30, and 90 days according to type of surgery

	Type of surgery			P*
	Pancreatoduodenectomy (*n* = 129)	Distal pancreatectomy (*n* = 82)	Total pancreatectomy (*n* = 26)	
**7 days after surgery**				< 0.001
None	80 (62%)	67 (81.7%)	15 (57.7%)	
Mild	20 (15.5%)	11 (13.4%)	2 (7.7%)	
Moderate	16 (12.4%)	3 (3.7%)	7 (26.9%)	
Severe	13 (10.1%)	1 (1.2%)	2 (7.7%)	
**30 days after surgery**				< 0.001
None	65 (50.4%)	64 (78.0%)	10 (38.5%)	
Mild	35 (27.1%)	13 (15.9%)	11 (42.3%)	
Moderate	17 (13.2%)	4 (4.9%)	4 (15.4%)	
Severe	12 (9.3%)	1 (1.2%)	1 (3.8%)	
**90 days after surgery**				0.016
None	84 (65.1%)	62 (75.6%)	13 (50.0%)	
Mild	28 (21.7%)	18 (22%)	12 (46.2%)	
Moderate	15 (11.6%)	2 (2.4%)	1 (3.8%)	
Severe	2 (1.6%)	0 (0%)	0 (0%)	

Values are *n* (%). *χ^2^ test or Fisher’s exact test.

At 7 days after surgery, 75 patients (31.6%) experienced diarrhoea. Among these, 44% described it as mild, 34.7% as moderate, and 21.3% as severe. Patients experiencing diarrhoea at this point had a median of 4 (i.q.r. 3–6) diarrhoeal episodes/day and a median stool consistency score of 6 (6–6) on the BSS. Regarding associated symptoms, 86.7% of patients with diarrhoea experienced urgency, 28% abdominal discomfort, and 21.3% faecal incontinence. In contrast, among patients without diarrhoea, urgency, abdominal discomfort, and faecal incontinence were reported in 0, 30.8, and 0% of patients, respectively. Statistical analysis showed that urgency (*P* < 0.001) and faecal incontinence (*P* = 0.002) were significantly more frequent among patients with diarrhoea, whereas abdominal discomfort did not differ significantly (*P* = 0.801). PERT use was reported by 89.3% of patients with diarrhoea (median dose 50 000 LU/day) compared with 55.1% of patients without (median dose 35 000 LU/day) (*P* < 0.001). Among those with diarrhoea, the rate of PERT use was 69.7% for those with mild diarrhoea, and 100% for those with moderate and severe diarrhoea. The mean(s.d.) QoL score in the overall cohort was 9.2(14.7). Patients with diarrhoea had significantly worse QoL scores (mean 29.2(9.9); *P* < 0.001). The mean QoL score was 21.0(4.6) for patients with mild diarrhoea, 31.4(5.9) for those with moderate diarrhoea, and 42.0(6.7) for patients with severe diarrhoea. The mean STIDAT score for the overall cohort was 1.6(1.8), whereas it was significantly higher in patients with diarrhoea (3.2(1.2); *P* < 0.001). The mean STIDAT score was 2.7(0.2), 4.2(0.5), and 5.9(0.9) for patients with mild, moderate, and severe diarrhoea, respectively. Regarding the incidence of diarrhoea at 7 days by type of surgery, it was present in 38.0% of patients after PD, 18.3% after DP, and 42.3% after TP (*P* < 0.001).

Outcomes 30 days after surgery

At 30 days after surgery, 98 patients (41.4%) reported diarrhoea (60.2% mild, 25.5% moderate, and 14.3% severe). Patients with diarrhoea had a median of 3 (i.q.r. 3–5) diarrhoeal episodes/day and a median stool consistency score of 6 (6–6) on the BSS, significantly higher than those without diarrhoea (*P* < 0.001). In patients with diarrhoea, urgency was reported by 52%, abdominal discomfort by 69.4%, and faecal incontinence by 18.4%. Among patients without diarrhoea, the same symptoms were reported in substantially lower proportions (urgency 0%, abdominal discomfort 21.0%, and incontinence 0%). Some 96.9% of patients with diarrhoea reported using PERT (median dose 80 000 LU/day), compared with 39.6% of patients without diarrhoea (median dose 10 000 LU/day) (*P* < 0.001). Some 23.4% of patients with diarrhoea used antidiarrhoeals in addition to PERT. The mean(s.d.) QoL score in the overall cohort was 11.0(13.8). Patients with diarrhoea had significantly worse QoL scores (mean 24.0(12.4); *P* < 0.001). The mean QoL score was 16.9(8.2) for patients with mild diarrhoea, 31.3(10.0) for those with moderate diarrhoea, and for 40.6(6.1) for those with severe diarrhoea. The mean STIDAT score was 1.8(1.6) in the overall cohort, and significantly higher in patients with diarrhoea (3.4(1.2); *P* < 0.001). Patients without diarrhoea had a mean STIDAT score of 0.6(0.2). Scores for those with mild, moderate, and severe diarrhoea were 2.6(0.4), 4.1(0.5), and 5.5(0.4), respectively. Regarding the incidence of diarrhoea at 30 days by type of surgery, it affected 49.6% of patients after PD, 22% after DP, and 61.5% after (*P* < 0.001).

Outcomes 90 days after surgery

At 90 days after surgery, the overall incidence of diarrhoea was 32.9%. Among these patients, 74.3% reported it as mild, 23.1% moderate, and 2.6% severe. Patients with diarrhoea had a median of 3 (i.q.r. 3–3) episodes/day and a median stool consistency score of 6 (6–6) on the BSS, both significantly higher than those without diarrhoea (*P* < 0.001). Among patients with diarrhoea, urgency was reported by 39.7%, abdominal discomfort by 51.3%, and faecal incontinence by 7.7%. Among patients without diarrhoea, the same symptoms were reported in substantially lower proportions (urgency 0.5%, abdominal discomfort 15.0%, and incontinence 0.5%). PERT use was reported by 94.9% of patients with diarrhoea (median dose 85 000 LU/day) *versus* 50.9% of those without (median dose 20 000 LU/day) (*P* < 0.001). Among patients with diarrhoea, 37.2% used antidiarrhoeals in addition to PERT. The mean(s.d.) QoL score in the overall cohort was 6.6(10.6). Patients with diarrhoea continued to have significantly worse QoL scores (mean 17.3(12.2); *P* < 0.001). The mean QoL score was 12.6(9.6) for those with mild, 30.4(7.4) for patients with moderate, and 37.5(3.5) for those with severe diarrhoea. The mean STIDAT score was 1.3(1.2) in the overall cohort, and significantly higher among those with diarrhoea (2.8(0.9); *P* < 0.001). The mean STIDAT score for patients with mild, moderate, and severe diarrhoea was 2.4(0.5), 4.0(0.4), and 5.0(0.4), respectively. Regarding the incidence of diarrhoea at 90 days by type of surgery, it was experienced by 34.9% of patients after PD, 24.4% after DP, and 50% following TP (*P* < 0.040).

### Predictors of moderate/severe postoperative diarrhoea

Univariable and multivariable logistic regression analyses were undertaken to identify predictors of moderate or severe diarrhoea (classified based on subjective impact on daily life) occurring at 30 and/or 90 days after operation (*Table 5*). In the univariable analysis, preoperative diarrhoea (OR 3.04, 95% confidence interval 1.45 to 6.39; *P* = 0.003), preoperative PERT use (OR 3.18, 1.51 to 6.69; *P* = 0.002), pancreatic ductal adenocarcinoma (PDAC) diagnosis (OR 3.84, 1.75 to 8.43; *P* = 0.001), vascular resection (OR 5.36, 2.30 to 12.49; *P* < 0.001), arterial divestment (OR 4.37, 1.49 to 12.80; *P* = 0.007) and FRS < 3 (OR 2.59, 1.14 to 5.90; *P* = 0.023) were significantly associated with the development of moderate-to-severe PPD, whereas undergoing DP compared with PD (OR 0.18, 0.06 to 0.48; *P* = 0.001) and ETS use (OR 0.33, 0.13 to 0.81; *P* = 0.016) were associated with a lower PPD risk.

**Table 5:** Univariable and multivariable logistic regression analysis of predictors of moderate/severe diarrhoea at 30 and/or 90 days after surgery

	Univariable analysis		Multivariable analysis	
	Odds ratio	*P*	Odds ratio	*P*
Age (per year)	0.99 (0.97, 1.02)	0.738		
Female sex	1.44 (0.74, 2.82)	0.280		
Preoperative weight loss	1.67 (0.86, 3.25)	0.125		
Preoperative diarrhoea	3.04 (1.45, 6.39)	0.003	1.15 (0.31, 4.26)	0.831
Preoperative PERT use	3.18 (1.51, 6.69)	0.002	2.01 (0.53, 7.53)	0.298
Preoperative antidiarrhoeal use	3.08 (0.50, 19.08)	0.225		
PDAC diagnosis	3.84 (1.75, 8.43)	0.001	2.64 (1.11, 6.29)	0.028
**Type of surgery**				
PD	1.00 (reference)		1.00 (reference)	
DP	0.18 (0.06, 0.48)	0.001	0.24 (0.08, 0.71)	0.010
TP	0.66 (0.23, 1.90)	0.447	0.77 (0.24, 2.43)	0.657
Vascular resection	5.36 (2.30, 12.49)	< 0.001	3.11 (1.21, 8.02)	0.018
Arterial divestment	4.37 (1.49, 12.80)	0.007	3.64 (1.10, 12.03)	0.034
Extended lymphadenectomy	2.22 (0.93, 5.29)	0.071	2.65 (0.97, 7.21)	0.056
Pylorus preservation (if PD)	1.54 (0.53, 4.50)	0.424		
FRS < 3 (if PD)	2.59 (1.14, 5.90)	0.023		
ETS (if PD)	0.33 (0.13, 0.81)	0.016		

Values in parentheses are 95% confidence intervals. PERT, pancreatic enzyme replacement therapy; PDAC, pancreatic ductal adenocarcinoma; PD, pancreatoduodenectomy; DP, distal pancreatectomy; TP, total pancreatectomy; FRS, fistula risk score; ETS, externalized transanastomotic stent.

In multivariable analysis, DP was independently associated with a lower risk of developing moderate/severe PPD compared with PD (OR 0.24, 0.08 to 0.71; *P* = 0.010). Conversely, PDAC diagnosis (OR 2.64, 1.11 to 6.29; *P* = 0.028), vascular resection (OR 3.11, 1.21 to 8.02; *P* = 0.018), and arterial divestment (OR 3.64, 1.10 to 12.03; *P* = 0.034), were independently associated with a higher risk of developing moderate-to-severe PPD at 30 and/or 90 days after surgery.

## Discussion

This prospective single-institution study has shown that more than one-third of patients undergoing pancreatectomy develop postoperative diarrhoea, which significantly impairs QoL even after correction for pancreatic insufficiency with the use of PERT. The development of PPD is more frequent in patients with PDAC and in those requiring vascular resections and arterial divestment, and should be underscored once present at discharge, as these patients represent a target for specific interventions aimed at reducing the detrimental impact of diarrhoea.

Although essential for treating pancreaticobiliary malignancies, pancreatic surgery often results in postoperative complications. A common yet scarcely explored issue is postoperative diarrhoea, which can be challenging and persistent, significantly affecting patient outcomes^15,37^. Its aetiology is multifactorial, involving varying degrees of EPI, altered nutrient absorption owing to anatomical changes, modified gut motility, and both neural and hormonal dysregulation. Additional mechanisms may include sympathetic and parasympathetic denervation following extended lymphadenectomy, disruption of enteroendocrine signalling after duodenal resection, microbiota alterations related to perioperative antibiotic exposure, BAM, and, in selected patients, the effects of adjuvant chemotherapy. Conceptually, PPD should be regarded as a functional sequela rather than a classical surgical complication. It primarily reflects long-term physiological adaptations to pancreatic resection—such as exocrine insufficiency, autonomic denervation, and bile acid or motility alterations—highlighting its chronic, multifactorial nature and the need for multidisciplinary long-term management^12,35,38,39^. PPD can severely impair postoperative recovery, hinder initiation of adjuvant therapy, cause malnutrition, and profoundly diminish QoL. Despite its prevalence and significant clinical impact, PPD often remains under-recognized, inadequately investigated, and is consequently managed suboptimally. In addition, owing to cultural and personal factors related to its reporting, many patients suffer from persistent symptoms without a formal diagnosis or targeted treatment plan^13,15,17^. The present prospective analysis recorded incidence rates of 31.6% at 7 days after surgery, peaking at 41.4% at 30 days, and persisting in 32.9% of patients at 90 days (*Fig. 1*). This incidence falls squarely within the broad range (20 to > 50%) cited in literature, confirming the overall high prevalence of PPD^13,15,38,40,41^. The timing of symptoms, peaking at 30 days after surgery, is also aligned with the timeline found in literature, with the greatest incidence of symptoms reported around 4–6 weeks after operation^13^. Of note, this timing coincides with the critical window for initiating adjuvant chemotherapy for many patients with pancreatic cancer, underscoring the detrimental clinical implications of uncontrolled diarrhoea. Although the overall incidence of PPD decreased by 90 days, its persistence in nearly one-third of patients emphasizes the chronic nature of this problem for a substantial subgroup of patients, extending well beyond the immediate recovery phase.

A key challenge in assessing and therefore treating PPD is the lack of a standardized definition and uniform assessment methods. To address this issue, a structured approach was employed in the present analysis, which was based on an adapted questionnaire derived from the validated STIDAT tool and the BSS, along with symptom reporting. Although adapting a tool requires caution as the questionnaire used here was not validated in this specific cohort, such an approach provides a more systematic way of capturing all symptom dimensions (frequency, consistency, urgency, incontinence) and QoL impact compared with unstructured questioning. These questionnaires offer a practical, relatively simple, and rapid means of investigating PPD. This structured approach not only facilitates early recognition of the condition but also allows tracking of its severity over time (as demonstrated by the 7-, 30-, and 90-day data), and may enable a more objective evaluation of treatment responses compared with unstructured clinical questioning. The severity of symptoms was classified as mild, moderate, or severe based on the impact on their daily lives reported by patients. Although this approach is subjective, it is indeed clinically relevant, reflecting the functional burden of symptoms. Moderate-to-severe diarrhoea affected a significant proportion of patients, particularly early on (56% of those with diarrhoea at 30 days), although the proportion of severe cases decreased over time. At 90 days, diarrhoea was reported in 32.9% of the total cohort; among these affected patients, 23.1% experienced moderate symptoms (corresponding to 7.6% of the overall cohort) (*Fig. 1*). Additionally, associated symptoms such as urgency, which affected over 85% of patients with PPD at 7 days, were common. Other symptoms like abdominal discomfort and incontinence affected more than 20% of patients at the same time point, further illustrating the overall burden of symptoms.

The present study has highlighted the significant negative impact of PPD on patients’ QoL. Those who experienced diarrhoea consistently reported significantly worse QoL scores than those without diarrhoea at all time points, with statistically significant differences observed both at 30 and at 90 days after surgery. On postoperative day 7, overall QoL scores were uniformly poor, reflecting the general burden of major pancreatic surgery. Nevertheless, the significantly worse scores among patients with diarrhoea suggest that, even in the early recovery phase, PPD may have an additional and measurable impact on patient well-being beyond the expected postoperative discomfort. Furthermore, there was a clear correlation between increasing subjective diarrhoea severity and worsening QoL scores, providing quantitative evidence for the functional impairment caused even by subjectively mild symptoms. The assessment of QoL was based on five specific questions derived from the STIDAT. Although this approach can be useful, it is less standardized than widely validated QoL questionnaires. For instance, general instruments such as the European Organization for Research and Treatment of Cancer QLQ-C30 are beneficial for comparing QoL across patients with cancer and include a question about diarrhoea. However, this instrument lacks the sensitivity needed to fully capture the range and impact of gastrointestinal symptoms specific to pancreatic surgery, such as those associated with EPI. Moreover, it does not objectively measure the incidence of diarrhoea, and is not pancreas-specific^42,43^. Because of the limitations of generic tools in capturing the specific details of PPD, the authors deemed it necessary to create an evaluation tool tailored to the objectives of the present research, focused on the frequency of symptoms, associated issues, and their impact on QoL.

Consistent with existing literature^13,35,44^, the incidence of PPD varied by surgical procedure. At 30 days, its incidence was higher after TP (61.5%) and PD (49.6%) compared with DP (22.0%). This trend generally persisted at 90 days (*Fig. 2*). The association between type of surgical intervention and incidence of PPD was statistically significant both at 30 days (*P* < 0.001) and 90 days (*P* = 0.042). Such higher rates after TP and PD likely reflect the near-universal development of significant EPI following these resections. The lower, though still notable, rate after DP aligns with the variable prevalence of EPI after such an operation^21,41,45^. The multivariable analysis reinforced this evidence, revealing an independent association between DP and a significantly lower risk of developing moderate-to-severe diarrhoea compared with PD (adjusted OR 0.24). Beyond diarrhoea, the type of surgical intervention also significantly influenced the incidence of other key gastrointestinal symptoms, most notably faecal urgency. At 30 days after surgery, urgency was reported more frequently after TP (38.5%) and PD (27.9%) than after DP (6.1%) (*P* < 0.001). This pattern persisted and remained statistically significant at 90 days. In contrast, the incidence of abdominal discomfort and faecal incontinence did not show an association with the type of surgical procedure either at 30 or 90 days after surgery. Although urgency seems to be linked to the extent of pancreatic resection, symptoms like abdominal discomfort and incontinence may be influenced by other factors, including microbiota alterations, infections, adhesions, gastrointestinal co-morbidities or other pre-existing conditions. Moreover, abdominal discomfort at 7 days should be interpreted with caution, as it may primarily reflect the immediate postoperative course, including surgical pain, delayed gastric emptying or transient bowel dysfunction, rather than diarrhoea-related symptoms.

The clinical importance of PPD underscores the need to predict its occurrence and gravity, and both vascular resection (OR 3.11) and arterial divestment (OR 3.64) were identified as independent predictors of subsequent moderate-to-severe diarrhoea. Vascular resections and divestments in pancreatic surgery are fully incorporated into the toolbox of modern cancer surgery nowadays. They often involve extensive dissection of vessels, potentially damaging the autonomic nerve plexuses surrounding major arteries. This damage is increasingly being recognized as a factor contributing to refractory, possibly neurogenic, diarrhoea^38,40^. Patients undergoing such cancer surgery and being discharged with ongoing PPD should be carefully targeted for dietary and therapeutic interventions. PDAC aetiology was also independently associated with PPD (OR 2.64), as a consequence of the frequent combination with pre-existing EPI due to long-standing ductal obstruction and/or pre-existing chronic pancreatitis. Resection of an already compromised gland could lead to more severe postoperative EPI, contributing to PPD. Similarly, a low intraoperative FRS (< 3), implying a hard pancreatic gland and a dilated duct (typical findings in head PDAC), was associated with an increased PPD risk in univariable analysis. For the opposite reasons, the selective placement of an ETS (used in high-risk patients, with a high FRS, and a functioning pancreas) to mitigate pancreatic fistula was associated with less frequent PPD. However, both associations (FRS and ETS) were not tested in multivariable analysis restricted to patients with PD only, owing to the small sample size. Interestingly, PPD also showed a significant association with POPF and chyle leaks. Although the lower rates of POPF in patients with diarrhoea are justified by the generally reduced pancreatic function, the increased rates of chyle leak suggest a shared pathophysiological basis, as both complications can develop from lymphatic dissection and disruption of autonomic nerve plexus inherent to pancreatic surgery.

PERT was used extensively among patients reporting PPD (reaching 97% at 30 days and 95% at 90 days), with mean doses increasing over the follow-up period. The mean PERT dose at discharge (58 000 LU/day) may appear lower than standard guideline recommendations; however, this reflects the early postoperative phase, characterized by limited oral intake and cautious dose initiation. Enzyme supplementation was progressively adjusted at follow-up according to individual dietary tolerance and symptom control. The relatively low PERT doses used in the present cohort may also partly reflect the current shortage of pancreatic enzyme formulations across Europe. This limited availability likely influenced real-world prescribing practices, potentially preventing optimal dose titration in some patients^46^. Despite the widespread and intensifying PERT use, PPD persisted in over 30% of the cohort at 90 days. This observation, along with the high prevalence of associated symptoms like urgency, and the link between vascular resection and severe diarrhoea, strongly suggests that EPI, while being a major contributor, is often not the sole cause of symptoms. Other factors such as BAM, SIBO, surgically induced dysmotility, or nerve injury likely play significant roles, contributing to the multifactorial nature of PPD^19,21,38,47^. The substantial use of antidiarrhoeal medications like loperamide further indicates that optimizing PERT alone is often inadequate, especially in patients with moderate-to-severe diarrhoea persisting at 90 days (over 70% of patients with moderate and 100% with severe diarrhoea used antidiarrhoeals). Non-responding patients should prompt consideration of, and potentially targeted investigation for, other contributing factors. Given the complexity of PPD, collaboration between surgeons, oncologists, dietitians, and pancreatic gastroenterologists is vital. The role of the gastroenterologist is pivotal, offering specialized diagnostics for non-EPI-related diarrhoea, and guiding advanced therapies beyond PERT.

The strengths of this study include its structured prospective design, the inclusion of different resection types, standardized data collection using questionnaires addressing symptoms and QoL, and longitudinal follow-up. However, there are limitations to consider. The study was conducted at a single centre, which may have restricted the generalizability of the findings. Additionally, the reliance on subjective patient assessments for diarrhoea severity, while clinically relevant, is less objective than sole use of standardized diarrhoea assessment tools (STIDAT scores). Another significant limitation of this study is the inability to differentiate systematically between diarrhoea caused by EPI and diarrhoea with other aetiologies, such as BAM, SIBO, surgically induced dysmotility, or nerve plexus dissection. Objective diagnostic testing (for example faecal elastase 1 measurement, 75-selenium homocholic acid taurine (SeHCAT) test, breath testing) was not included in this protocol and should be incorporated in future prospective studies. This prevents definitive attribution of the underlying causes of PPD in individual patients and limits interpretation of PERT non-response. Additionally, the potential influence of the underlying pathology (for example malignancy *versus* benign disease) or the effect of neoadjuvant therapy was not explored extensively, but could also affect postoperative bowel function. Finally, longer follow-up is needed to assess the true lifelong burden of diarrhoea after pancreatectomy, as the symptoms may decrease or even disappear beyond the first 3 months after surgery.

This prospective study has highlighted the high incidence of PPD, which exceeds 40% at 30 days after surgery, and its significant impact on QoL, particularly after PD and TP. Despite the widespread use of PERT, diarrhoea remains a persistent issue, indicating a multifactorial cause. Factors significantly associated with a higher risk of moderate-to-severe diarrhoea include PDAC diagnosis and the need for vascular resection and/or arterial divestment, whereas DP was associated with a lower risk than PD. These results underline the critical need for proactive, systematic assessment using standardized tools, alongside recognition of the diverse potential underlying causes (including EPI, BAM, SIBO, gastrointestinal dysmotility). A multidisciplinary approach is essential for managing this complex complication, focusing on early identification, PERT optimization, and investigation of different possible aetiologies.

## Supplementary Material

zrag017_Supplementary_Data

## Data Availability

The data are not publicly available owing to privacy restrictions, but are available from the corresponding author upon reasonable request.
