# Supplementary material for: Postpancreatectomy diarrhoea: prospective, single-centre longitudinal analysis of incidence, risk factors, management, and impact on quality of life
Source: BJS Open. 2026 Mar 24;10(2):zrag017. doi: 10.1093/bjsopen/zrag017 (PMC13010069; doi:10.1093/bjsopen/zrag017)
Supplement: zrag017_Supplementary_Data [file zrag017_supplementary_data.docx]

**Title:**

Post-pancreatectomy diarrhea: incidence, risk factors, management, and impact on quality of life.

A prospective, single-center longitudinal analysis

**Authors:**

Giampaolo Perri^1,^ Livia Zornetta^2^, Riccardo Pellegrini^1^, Pietro Rigo^1^, Nicola Canitano^1^, Domenico Bassi^1^, Patrizia Burra^2^, Umberto Cillo^1^, Giovanni Marchegiani^1^

^1^Hepato-pancreato-biliary and Liver Transplant Surgery Unit, Department of Surgical, Oncological and Gastroenterological Sciences (DiSCOG), University of Padua (Padua, Italy)

^2^Gastroenterology and Multivisceral Transplant Unit Department of Surgical, Oncological and Gastroenterological Sciences (DiSCOG), University of Padua (Padua, Italy)

**Corresponding author**

Giovanni Marchegiani, MD, PhD

Chirurgia Epato-Bilio-Pancreatica e dei Trapianti di Fegato

Dipartimento di Scienze Chirurgiche Oncologiche e Gastroenterologiche - DISCOG

Università di Padova

Via Giustiniani 2, 35128 Padova

Email: giovanni.marchegiani@unipd.it

**ORCID ID** [**https://orcid.org/0000-0002-6824-4533**](https://orcid.org/0000-0002-6824-4533)

**Twitter** @Gio_Marchegiani

**Supplementary Materials - Index**

| **Supplementary Figures and Tables** |  |
| --- | --- |
| Supplementary Table S1 | *pag. 2* |
| Supplementary Table S2 | *pag. 3* |
| Supplementary Figure S1 | *pag. 4* |
| Supplementary Figure S2 | *pag. 4* |
| **Supplementary Appendixes** |  |
| Supplementary Appendix 1 | *pag. 5* |
| Supplementary Appendix 2 | *pag. 6* |

**Supplementary Tables and Figures**

**Table S1: STROBE Checklis****t**

|  | Item No. | Recommendation | | Page  No. |
| --- | --- | --- | --- | --- |
| **Title and abstract** | 1 | (*a*) Indicate the study’s design with a commonly used term in the title or the abstract | | 1 |
|  |  | (*b*) Provide in the abstract an informative and balanced summary of what was done and what was found | | 2 |
| Introduction | | |  | |
| Background/rationale | 2 | Explain the scientific background and rationale for the investigation being reported | | 3 |
| Objectives | 3 | State specific objectives, including any prespecified hypotheses | | 4 |
| Methods | | |  | |
| Study design | 4 | Present key elements of study design early in the paper | | 4 |
| Setting | 5 | Describe the setting, locations, and relevant dates, including periods of recruitment, exposure, follow-up, and data collection | | 4 |
| Participants | 6 | (*a*) *Cohort study*—Give the eligibility criteria, and the sources and methods of selection of participants. Describe methods of follow-up | | 4-5 |
| Variables | 7 | Clearly define all outcomes, exposures, predictors, potential confounders, and effect modifiers. Give diagnostic criteria, if applicable | | 4-5 |
| Data sources/ measurement | 8 | For each variable of interest, give sources of data and details of methods of assessment (measurement). Describe the comparability of assessment methods if there is more than one group | | *4-5* |
| Bias | 9 | Describe any efforts to address potential sources of bias | | 4-5 |
| Study size | 10 | Explain how the study size was arrived at | | 4 |
| Quantitative variables | 11 | Explain how quantitative variables were handled in the analyses. If applicable, describe which groupings were chosen and why | | 6 |
| Statistical methods | 12 | (*a*) Describe all statistical methods, including those used to control for confounding | | 6 |
|  |  | (*b*) Describe any methods used to examine subgroups and interactions | | 6 |
|  |  | (*c*) Explain how missing data were addressed | | 6 |
|  |  | (*d*) *Cohort study*—If applicable, explain how loss to follow-up was addressed | | 6 |
| **Results** |  |  | |  |
| Participants | 12 | (a) Report numbers of individuals at each stage of study—eg numbers potentially eligible, examined for eligibility, confirmed eligible, included in the study, completing follow-up, and analysed | | 6-9 |
|  |  | (b) Give reasons for non-participation at each stage | | 6-9 |
|  |  | (c) Consider use of a flow diagram | | Figure S1 |
| Descriptive data | 14* | (a) Give characteristics of study participants (eg demographic, clinical, social) and information on exposures and potential confounders | | 6-9 |
|  |  | (b) Indicate number of participants with missing data for each variable of interest | | 6-9 |
|  |  | (c) *Cohort study*—Summarise follow-up time (eg, average and total amount) | | 6-9 |
| Outcome data | 15* | *Cohort study*—Report numbers of outcome events or summary measures over time | | 6-9 |
| Main results | 16 | *(a*) Give unadjusted estimates and, if applicable, confounder-adjusted estimates and their precision (eg, 95% confidence interval). Make clear which confounders were adjusted for and why they were included | | 6-9 |
|  |  | (*b*) Report category boundaries when continuous variables were categorized | | 6-9 |
|  |  | (*c*) If relevant, consider translating estimates of relative risk into absolute risk for a meaningful time period | | 6-9 |
| Other analyses | 17 | Report other analyses done—eg, analyses of subgroups and interactions, and sensitivity analyses | | 6-9 |
| **Discussion** |  |  | |  |
| Key results | 18 | Summarise key results with reference to study objectives | | 10-13 |
| Limitations | 19 | Discuss limitations of the study, taking into account sources of potential bias or imprecision. Discuss both the direction and magnitude of any potential bias | | 13 |
| Interpretation | 20 | Give a cautious overall interpretation of results considering objectives, limitations, multiplicity of analyses, results from similar studies, and other relevant evidence | | 9-13 |
| Generalisability | 21 | Discuss the generalisability (external validity) of the study results | | 13 |
| **Other information** |  |  | |  |
| Funding | 22 | Give the source of funding and the role of the funders for the present study and, if applicable, for the original study on which the present article is based | | 9-13 |

**Table S2: Complication rates by Postoperative Diarrhea Incidence at 30 days**

|  | **30 days** | | | |
| --- | --- | --- | --- | --- |
| **Complication** | **All paitents**  **(N= 239)** | **Patients w/diarrhea**  **(N= 98)** | **Patients**  **w/out diarrhea**  **(N=141)** | **p-value** |
| **POPF, No. (%)** | 45 (19.0%) | 10 (10.2%) | 35 (25.2%) | **0.004** |
| **PPAP, No. (%)** | 23 (9.7%) | 9 (9.1%) | 14 (10.1%) | 0.808 |
| **PPH, No. (%)** | 57 (24%) | 25 (23.4%) | 32 (33.6%) | 0.615 |
| **DGE, No. (%)** | 32 (13.5%) | 9 (9.1%) | 23 (16.5%) | 0.111 |
| **Chyle leak, No. (%)** | 16 (6.8%) | 13 (13.3%) | 3 (2.2%) | **<0.001** |
| *Abbreviations: POPF, postoperative pancreatic fistula; PPAP, post-pancreatectomy acute pancreatitis; PPH post-pancreatectomy hemorrhage; DGE, delayed gastric emptying.* | | | | |

**Figure S1: Study Flow Chart**


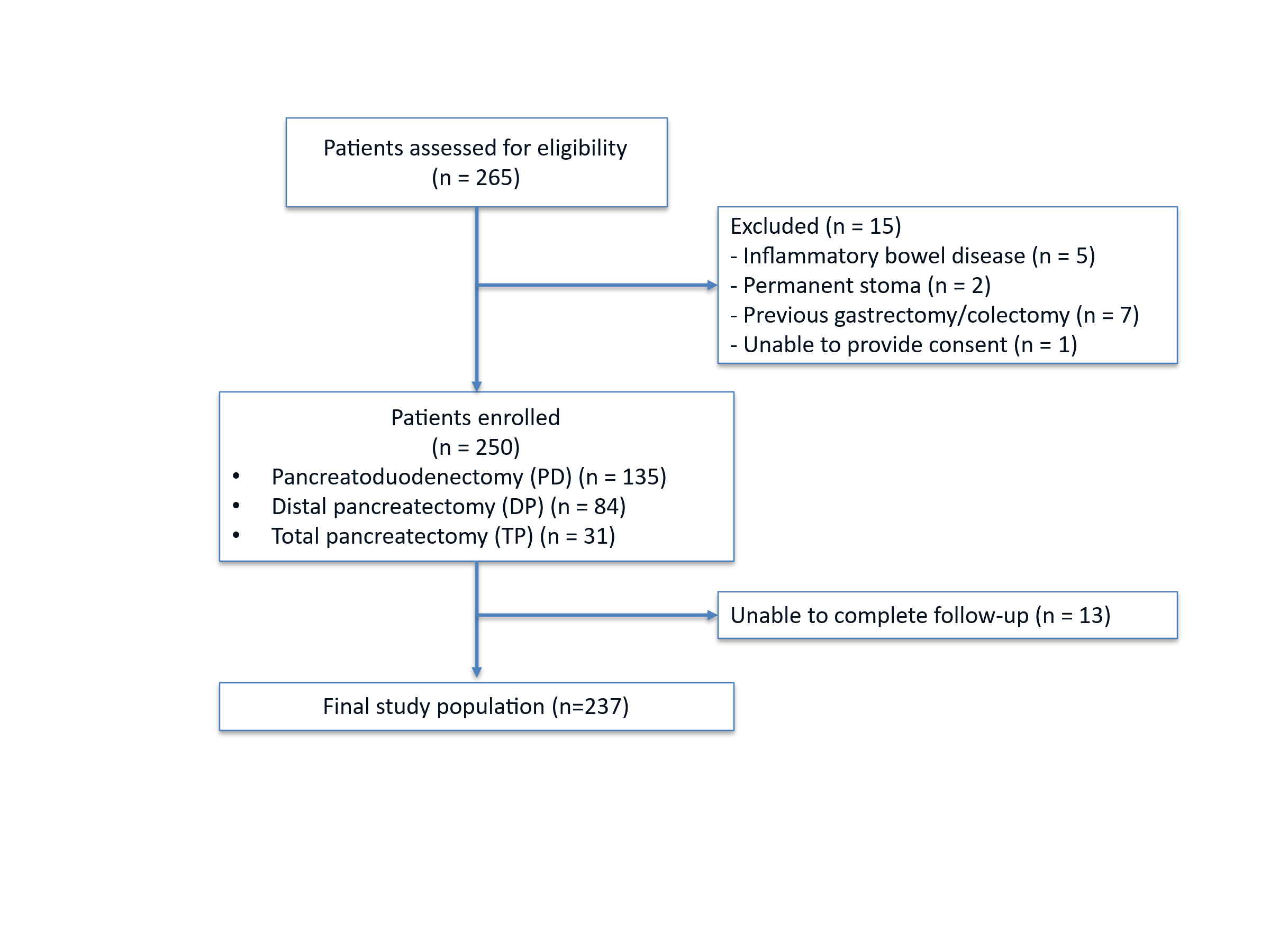


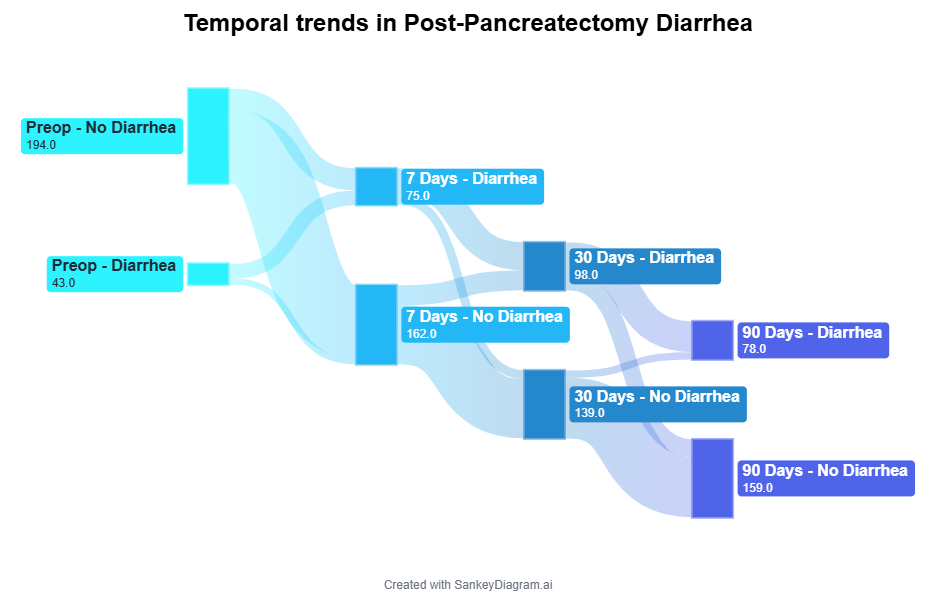
**Figure S2: Sankey diagram illustrating the evolution of diarrhea status from the preoperative baseline to 90 days postoperatively.**

**Supplementary Appendixes**

**Appendix 1: STIDAT-derived questionnaire**

**Onset and duration**

1. In the last 7 days, did you experience any diarrhea? (If no, skip to question 3).
   1. If yes, how would you rate your diarrhea at your wrost?

Minimal diarrhea Moderate diarrhea Severe diarrhea

**Stool frequency**

1. On average, how many times did you have diarrhea per day in the last 7 days?

____per day

1. On average, how many times did you pass normal stool per day in the last 7 days? ____per day

**Diarrhea-associated symptoms**

1. In the last 7 days, have you felt you suddenly had pass a stool?
2. In the last 7 days, did you have any abdominal discomfort?
3. In the last 7 days, were there times when you did not make it to the bathroom for a bowel movement?

**Self-Treatment**

1. Please, skip the question if you do not have diarrhea. This question patients to medications that you may have used in the last 7 days to help your diarrhea. Please complete the following table.

| Medication | Did you use the medication? | Amount used in total | Did it help your diarrhea |
| --- | --- | --- | --- |
| CREON  (Pancreatic enzyme) | YES NO |  | YES NO |
| IMODIUM (Loperamide) | YES NO |  | YES NO |

**Impact on quality of life**

1. Rank how much your bowel habits in the last 7 days have affected your ability to perform work or daily activities of living.

| 0 | 1 | 2 | 3 | 4 | 5 | 6 | 7 | 8 | 9 | 10 |
| --- | --- | --- | --- | --- | --- | --- | --- | --- | --- | --- |

No impact Extreme impact

1. Rank how much your bowel habits in the last 7 days have affected your energy level.

| 0 | 1 | 2 | 3 | 4 | 5 | 6 | 7 | 8 | 9 | 10 |
| --- | --- | --- | --- | --- | --- | --- | --- | --- | --- | --- |

No impact Extreme impact

1. Rank how much your bowel habits in the last 7 days affected your mood.

| 0 | 1 | 2 | 3 | 4 | 5 | 6 | 7 | 8 | 9 | 10 |
| --- | --- | --- | --- | --- | --- | --- | --- | --- | --- | --- |

No impact Extreme impact

1. Runk how much your diarrhea has affected your family life.

| 0 | 1 | 2 | 3 | 4 | 5 | 6 | 7 | 8 | 9 | 10 |
| --- | --- | --- | --- | --- | --- | --- | --- | --- | --- | --- |

No impact Extreme impact

1. Rank how much your diarrhea has affected your social life.

| 0 | 1 | 2 | 3 | 4 | 5 | 6 | 7 | 8 | 9 | 10 |
| --- | --- | --- | --- | --- | --- | --- | --- | --- | --- | --- |

No impact Extreme impact

**Appendix 2. Scoring system**

| **Components** | **Proportion explaining diarrhea** | **Score calculation** |
| --- | --- | --- |
| Factor 1. Patient’s perception of diarrhea   - Presence of diarrhea - Severity of diarrhea - Presence of urgency | - 0.193 - 0.529 - 0.048 | N^1^ x 0.193  N^2^ x 0.529  N^1^ x 0.048 |
| Factor 2. Bowel movement frequency   - Number of bowel movements - Number of diarrhea episodes - Medication use - Quality of life | - 0.050 - 0.161 - 0.060 - - 0.048 | N^3^ x 0.050  N^3^ x 0.161  N^1^ x 0.060  Avarage N _1-5_  x – 0.048 |
| Factor 3. Fecal Incontinence   - Presence of fecal incontinence | - 0.016 | N^1^ x 0032 |
| Factor 4. Abdominal symptoms.   - Presence of abdominal spasm - Presence of abdominal discomfort | - 0.032 - 0.031 | N^1^ x 0.032  N^1^ x 0.031 |
| TOTAL |  | Sum of component scores +0.48^*^ |

N^1^: yes = 1 no = 0

N^2^: no = 0, mild = 1, moderate = 2, severe = 3

N^3^: an integer of n≥0

*adjustment factor.
